# Utilization of Antenatal Care and Skilled Birth Delivery Services in Sub-Saharan Africa: A Systematic Scoping Review

**DOI:** 10.3390/ijerph21040440

**Published:** 2024-04-03

**Authors:** Monsurat A. Lateef, Desmond Kuupiel, Gugu G. Mchunu, Julian D. Pillay

**Affiliations:** 1 Faculty of Health Sciences, Durban University of Technology, Durban 4001, South Africa; desmondk@dut.ac.za (D.K.); gugum6@dut.ac.za (G.G.M.); pillayjd@dut.ac.za (J.D.P.); 2 Discipline of Public Health Medicine, School of Nursing and Public Health, University of KwaZulu-Natal, Durban 4001, South Africa

**Keywords:** antenatal care, skilled birth delivery, utilization services, sub-Saharan Africa, systematic scoping review

## Abstract

Daily, the number of women who die around the world reaches an average of 800; these deaths are a result of obstetric complications in pregnancy and childbirth, and 99% of these deaths occur in low- and middle-income countries. This review probes the use of antenatal care (ANC) and skilled birth delivery (SBD) services in sub-Saharan Africa (SSA) and highlights research gaps using Arksey and O’Malley’s methodological approach. The screening of abstracts and full text was carried out by two independent authors who ensured the eligibility of data extraction from the included articles. An exploration of the data was undertaken with descriptive analyses. In total, 350 potentially eligible articles were screened, and 137 studies were included for data extraction and analysis. From the 137 included studies, the majority were from Ethiopia (*n* = 40, 29.2%), followed by Nigeria (*n* = 30, 21.9%). Most of the studies were published between 2019 and 2023 (*n* = 84, 61%). Significant trends and challenges with ANC and SBD services emerged from the studies. It is revealed that there are wide gaps in the utilization of ANC and SBD services. Policy attention, intervention strategies to improve access, resources, rural–urban disparity, and women’s literacy are recommended to improve the utilization of ANC and SBD services in SSA countries.

## 1. Introduction

Maternal mortality is a global health priority and an acceptable key indicator of global development [1]. It reflects the whole national health system and its pros and cons [2]. Globally, approximately 287,000 women died during and following pregnancy and childbirth in 2020, where 95% of these avoidable maternal deaths occurred in low- and middle-income countries [3]. 

One of the important measures of a country’s social and economic development is maternal mortality [2,4]. It is a very important health indicator and very crucial in determining other socio-cultural, political and transparency disparities [2]. Sub-Saharan Africa alone accounted for 70% (202,000 out of 287,000) of maternal deaths [3], making maternal mortality unacceptably high among women of reproductive age. For instance, 1 out of 5300 women die in a high-income country, compared to 1 out of 49 women who lose their lives during pregnancy or after childbirth [3] in low- and middle-income countries. These stark figures demonstrate the urgent need to reduce maternal mortality in low- and middle-income countries. 

The countdown to 2030 causes us to take a cursory look at the Sustainable Development Goal (SDG) 3.1, which envisions a reduction in maternal mortality to less than 70 per 100,000 livebirths and neonatal mortality to less than 12 per 1000 live births [5]; this information from the United Nations predicts a reduction of almost two thirds when compared to the predecessor of the SDGs known as the MDGs (millennium development goals) 5 [5]. Even though considerable progress has been made globally since the inception of the SDGs era in 2016, it is clear that many SSA countries are still lagging behind [6,7] and further improvements are needed. 

It is pertinent to note that the ability to meet with expectations of sustainable development goals 3.1 can save the lives of about 1.6 million [8] expected mothers. Research proves that about three-quarters of maternal deaths are traced to pregnancy-related complications; these complications could have been avoided if proper reproductive health treatments were accessible and utilised during pregnancy [3,9,10,11]. Understanding the positive role of antenatal care (ANC) and skilled birth delivery (SBD) services in achieving the SDG 3.1 target is crucial. The most salient component of reproductive health is ANC [12,13] and the provision of access to healthcare facilities during pregnancy with a view to ensuring complete interventions that are critical to the health of the mother and that of the newborn [14]. Furthermore, pregnant women could be recipients of vitamin supplements, eclampsia, and tetanus vaccines [15,16]. In addition, the access and utilization of ANC services allows for the good option of HIV testing and prescribing medication in order to avoid HIV transmission from the mother to the child [16]. In areas where malaria is endemic, pregnant women are provided with mosquito nets containing insecticide to prevent fatal illness [16]. The SBD service is when pregnant women deliver one or more infants at the end of pregnancy, which occurs in the health facility under the supervision of a midwife, nurse, or medical doctor [17]. The adequate usage of antenatal services (ANC) reduces maternal mortality, and previous studies have proven this [18,19].

The ultimate goal of ANC is to promote the health and wellbeing of the mother and infant during pregnancy through regular check-ups by doctors or midwives.

The World Health Organization (WHO) recommended that ANC be initiated within the first trimester of pregnancy with at least four visits and ideally eight visits [20,21]. These visits provide essential preventive care based on the early detection of complications such as anaemia, pre-eclampsia, and gestational diabetes that may occur during pregnancy, and provide treatment and necessary intervention before serious complications occur [13,22]. However, studies demonstrated that certain obstacles, including cultural attitudes, scarce health facilities, a lack of health workers and even the absence of knowledge of ANC treatments, are major key concerns for pregnant women and their health status. Current global evidence reports that 90% of women use ANC services at least once, and only 60% use ANC services at least four times, which is the minimum recommended by WHO. However, only 49% of women in SSA utilised ANC services at least four times [21]. Although previous studies have examined the utilization of ANC from a country-specific basis [21,23], for example, in Ghana, Dickson et al. reported that 88% of women utilise ANC services. Based on their finding, maternal education, wealth status, place of residence, parity, and ethnicity all affected the use of ANC. Adedokun et al. reported the determinant of adequate utilization of maternal health services in Nigeria. Their findings also revealed media exposure, education, marital status, employment status, obtaining permission to use health services, and distance to health facilities were determinant factors for the sufficient use of maternal health services.

Thus, to the best of our knowledge, there have been few studies to date that have systematically investigated how the service utilization of ANC and SBD services helps reduce maternal mortality. Many studies focused on access to health services, quality of care and even the determinants of maternal health services. It is imperative to conduct a systematic analysis of the area by reviewing the existing literature on service utilization to identify gaps and a view to developing strong evidence. This could then be used to inform policy making and decision making and to contribute to the development of better strategies to address existing challenges. It can also help to identify potential solutions and develop strategies to implement them. Hence, the objective of this scoping review is to explore the utilization of ANC and SBD services in SSA and identify research gaps.

## 2. Materials and Methods

Transparency, consistency, and clarity in every phase of a scoping review process are of the utmost importance to ensure that the findings are robust, comprehensive, and reliable [24]. As such, this study made use of the methodological framework outlined by Arksey and O’Malley and refined by Levac in [24,25]. This framework enabled us to review the literature on the utilization of ANC and SBD services in SSA and identify research gaps in the literature, as well as draw meaningful conclusions about the existing evidence base systematically and rigorously. The Arksey and O’Malley methodological framework includes the following essential steps:

(a) The identification of the research question;
(b) The identification of relevant studies from electronic databases;
(c) The selection of a study process;
(d) The charting of data;
(e) Collating, summarising and reporting the results.

**Identifying the research question**

The research question was as follows: What research evidence exists on the utilization of ANC and SBD services in SSA over the past decade? 

To ensure the relevance of this research question, the population, concept, and context (PCC) framework [26] as part of this study’s eligibility criteria was employed [26] (Table 1).

**Identification of relevant studies from electronic databases**

Relevant papers addressing the research objective were searched. In order to achieve this study’s objective, five electronic databases, including PubMed, SCOPUS, Web of Science, Google Scholar, and the WHO African Index, were undertaken using the PCC framework as a guide to retrieve articles published between the years 2012 and 2023. In addition to database searches, the relevant literature was manually searched from the eligible reference list of the included studies. In this stage of the search, no language, date or publication-type filters were applied. All search results were imported into an EndNote Library X20 that was created to manage all the citations efficiently. For the database searches, a search strategy in collaboration with an information scientist was developed to ensure the inclusion of all relevant keywords such as “Antenatal care”, “Skilled delivery”, “Postnatal care”, “Child health”, “Maternal health”, “Utilization”, and “Africa”. A Boolean operator was employed (AND/OR) alongside Medical Subject Heading (MeSH) terms to refine our search string (Supplementary Materials has a detailed search strategy). The syntax was adjusted based on each database’s requirements.

**Selection of study process**

A selection tool based on the inclusion criteria was developed using Google Forms and was then pilot-tested by two reviewers (MA and DK) using the 10 titles and abstracts to ensure its accuracy. A duplicate search was then conducted using the “Find Duplicate” function in the EndNote library to identify any duplicate items within the library. This helped us to quickly identify and remove all duplicates from the library. Two reviewers (MA and DK) independently conducted the study screening process to categorise titles and abstracts into two ‘inclusion’ and ‘exclusion’ groups. All discrepancies in responses during this screening stage were resolved by consensus and discussion. The full-text articles of all titles and abstracts that met the inclusion criteria during the initial screening phase were obtained and then screened independently by the two reviewers following the eligibility criteria as a guide. Where a consensus could not be reached between MA and DK, a third reviewer (JDP) was consulted to resolve any discrepancies. The PRISMA flow diagram was used to document the article selection process, ensuring transparency and accountability [27].

**Quality appraisal**

The Mixed Method Quality Appraisal Tool (MMAT) is based on a set of standardised criteria that evaluate the quality of the study based on the research question, design, data collection, data analysis, and presentation of the results. The evaluation of this methodological quality was investigated using MMAT version 2018 [28] and the risk of bias in the included studies. The criteria are based on widely accepted research guidelines. The tool was employed to evaluate the suitability of the study’s objective, the appropriateness of the study design, participant recruitment methods, data collection methods, data analysis techniques, and the presentation of findings/results. To determine the quality of the studies, a quality score based on established criteria was assigned as follows: a score of 50% indicated low quality, 51–75% indicated average quality, and 76–100% indicated high quality. It is crucial to conduct this rigorous assessment in order to identify any gaps in research. The quality appraisal was conducted independently by two reviewers (MA and DK), and any disagreements were resolved by a third reviewer (JDP).

**Charting the data**

The extraction of the data was performed using a spreadsheet, and the testing was conducted using 10 of the incorporated studies to confirm that it captured all the important data for this review. This pilot was used to identify any potential data gaps or other issues that could arise in extracting data from the included studies. It also allowed the team to refine the Excel spreadsheet to ensure it captured all the necessary data. Following the pilot test, all feedback was carefully considered, and the data extraction form was adjusted accordingly. All relevant data were extracted from the full texts of the studies by two independent reviewers (MA and DK). The utilization of both inductive and deductive approaches was used for data extraction [29]. The study characteristics that were extracted are as follows: year of publication, study title, aim/objective, country, study design and study population. The findings of this study in terms of ANC and SBD utilization services were also carefully extracted.

**Collating, summarising, and reporting the results**

The data extraction process utilised a hybrid approach combining inductive and deductive reasoning [29]; this allowed for more accurate data analysis and better results. In this process, a thorough analysis of the extracted information was conducted to identify patterns, themes, and trends in the existing research evidence concerning ANC and SBD utilization services in SSA. Using a narrative format, the extracted data were compiled and analysed to provide a more comprehensive view of the data. To present our findings in a comprehensive manner, descriptive analysis and narrative synthesis were employed. Descriptive analysis provides a detailed overview of the data, and narrative synthesis organises the findings into a coherent flow. The study outcomes included a comprehensive review of research and evidence on ANC and SAD utilization services in SSA. The findings of this study were reported by utilising the preferred reporting items for systematic reviews and meta-analyses extension for the scoping reviews (PRIS-MA-ScR) checklist [30].

## 3. Results

### 3.1. Study Selection

**Figure 1:**
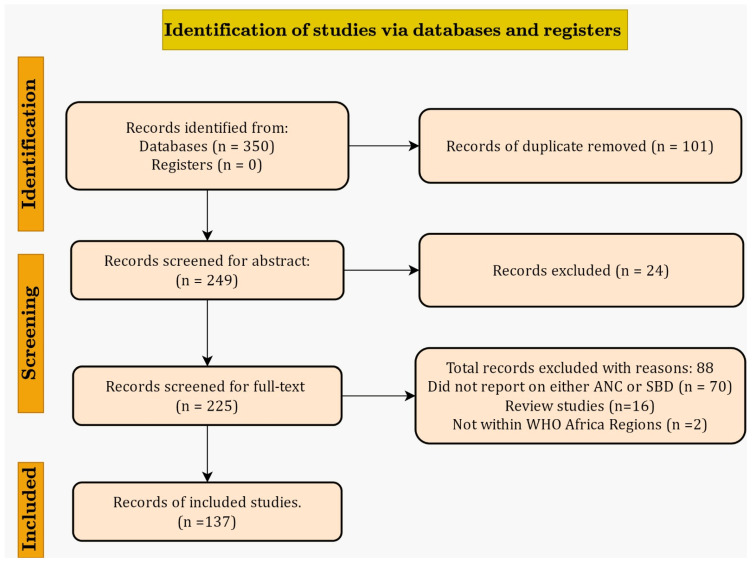
Adapted PRISMA flow 2020 diagram.

The authors screened 350 potentially eligible titles and abstracts across five databases. After excluding duplicates and those that did not meet our eligibility criteria, 137 studies were included for data extraction and analysis (Figure 1). Supplementary Materials presents a list of the documents that were not considered for the full-text screening phase.

#### Characteristics and Quality Appraisal of the Included Studies

From the 137 included studies, the majority were from Ethiopia (*n* = 40, 29.2%), followed by Nigeria (*n* = 30, 21.9%), and most of the studies were published between 2019 and 2023 (*n* = 84, 61%). Most of the included studies employed a cross-sectional study design (*n* = 119, 86.8%) and involved pregnant women and postnatal mothers (*n* = 124, 90.5%). The mean quality score ± SD of the 137 included studies was 88% ± 14. Table 2 shows details on the characteristics and quality appraisal of the included studies.

### 3.2. Study Findings

#### 3.2.1. Determinant of ANC and SBD Services

There have been several studies exploring the positive factors associated with ANC and SBD service utilization (Table 3). The findings from these studies indicated significant positive determinants and factors. Afaya et al., Amu et al., and Ameyaw et al. in Ghana, all reported on how awareness, education about reproductive age and being insured with the National Health Insurance Scheme adequately increase ANC visits among women by four or more times [97,123,125]. Geda et al.’s study in Ethiopia reported that female education, parity, residing in affluent households and polygamous families indicated positive influences on ANC service utilization [47]. Dimbuen et al.’s study in Congo, Ghana and Nigeria reported that households’ status, education, and access to health facilities were positive factors associated with ANC and SBD services utilization [131]. Afework et al. and Tiruneh et al. studies in Ethiopia identified a significant association between pregnant women visited by Health Extension Workers and four ANC attendances [33,62]. The ensuing factors have been pointed out as contributors to an increase in the utilization of ANC and SBD services: encouraging females/girls’ education [71,113], peer influence, spousal involvement/support in MHC partner education [58,71], partner educator [113,172], education monitoring strategies, unpleasant pregnancy history, employment, media, religion, and pregnancy preparedness. In Uganda, Guinea, Malawi and Ethiopia, studies in these aforementioned countries by Atuhaire et al., Atuoye et al., Stewart et al., and Tareke et al. discovered that having decision-making power to visit a health facility, financial independence and easy access to the health facilities were directly linked with ANC and SBD utilization services [56,119,164,180].

Alemayehu et al., Atuoye et al., Shibre et al., Yeneneh et al. and Tareke et al.’s findings in Ethiopia, Uganda, Guinea, Ethiopia and Ethiopia, respectively, indicated that living in urban areas, exposure to the media, and gravida and para were significantly associated with ANC 4+ utilization services [34,56,67,119,146]. Adewemimo et al., Alhassan et al. and Rosser et al.’s studies in Nigeria, Ghana and SSA reported on intervention to improve the majority of the health system, with an increase in frontline health staff and retention strategies, as well as investments in universal coverage to increase ANC and SBD utilization services [71,124,176]. Atake, 2018 in Togo, reported that a significant number of migrant mothers utilised ANC services in Togo compared to non-migrant women due to health insurance, which provides more financial protection to migrant mothers [161]. Atuhaire et al., Semagn, Rai et al., Birmeta et al., Adedokun et al. and Tesfaw et al.’s studies in Uganda, Ethiopia, Malawi, Ethiopia, SSA and Ethiopia, respectively, highlighted specific demographic and socio-economic factors such as age, educational status, short distance to a health facility, wealth index, husband approval, availability of health workers, and desire for pregnancy to increase early ANC utilization [40,53,59,94,166,180]. Birmeta et al. in Ethiopia identified knowledge about pregnancy danger and signs of complications [68] with ANC visits. Owiti et al.’s study in Kenya highlighted perceptions about the health facility, such as living within its proximity, having a support group and a short waiting time before being examined by the doctor, as impacting ANC utilization [107]. There is growing evidence that ANC and SBD utilization services have specific positive factors that need improvement for the upscale of ANC and SBD service utilization, which will inevitably influence achieving the SDG 3.1 target.

#### 3.2.2. The Prevalence of Low ANC Attendance and Associated Factors

Several studies have highlighted the prevalence rates of low ANC visits and factors associated with the low utilization of services in many SSA countries (Table 4). Abimbola et al.’s study in Nigeria reported on the following: lack of money, poor accessibility of healthcare services, long distance from the health facility, long waiting time, poor attitudes of health workers and no permission from husbands [92]. The long distance to the health facility and the cost of health services were among the leading challenges that were reported by several studies from different countries to undermine the utilization of ANC and SBD services. Kpienbaareh et al. and Nuamah et al.’s studies in Rwanda and Ghana reported on the association between women with low/no knowledge of pregnancy complications and the utilization of ANC services within the first trimester [129,133]. These aforementioned women were unlikely to complete the WHO-recommended minimum of eight visits.

Several studies have reported on the low utilization of ANC visits and SBD services due to many factors, such as socio-economic factors, place of residence, poor access to health facilities, lack of transportation, poor knowledge, and literacy. Arefaynie et al.’s findings in Ethiopia highlighted how living in rural areas, poverty, lack of education and single motherhood are associated with a low number of ANC visits [36]. Akinyemi et al. and Olayinka et al.’s studies in Nigeria identified that the death of a preceding child and/or previous bad obstetrics history was associated with a low rate of ANC and SBD service utilization [74,88]. Amouzou et al.’s study indicated that the COVID-19 pandemic had a negative impact on the service utilization of ANC-1 and ANC-4 in most SSA countries [181]. Kim et al. Langa and Bhatta, Uldbjerg et al. and Rurangirwa et al.’s studies in Senegal, Tanzania, Uganda and Rwanda, respectively, reported that women and adolescents with an unplanned pregnancy, limited knowledge, a poor social support system and perceived harshness of healthcare providers saw these factors as reasons for the low utilization of ANC [116,135,156,172]. Shatilwe et al.’s study in Namibia, Ayodo et al.’s study in Kenya and Uidbjerg et al.’s study in Uganda found that poor government infrastructure and poor quality of service delivery contributed to the poor uptake of ANC services [100,116,154]. Dadi et al.’s study in Ethiopia reported on a shortage of skilled personnel and equipment supplies as a major obstacle to maternal health service utilization [42]. Hitimana et al.’s study in Rwanda also revealed a low educational level and being single/unmarried as an issue affecting ANC [132], Okonofua et al.’s study in Nigeria reported no access to media, the absence of an educated partner, and high cost of MHS were significantly associated with poor utilization services [87]. Kawungezi et al. and Shatilwe et al. studies in Uganda and Namibia, respectively, reported on distance to the health facility, husband’s decisions/support, and the availability, the involvement of traditional birth attendance (TBA), wrong opinions during pregnancy about ANC, and poor financial support as constraints to adequate ANC service utilization [114,154].

A study conducted in Senegal by Kim et al., Kim et al. in Malawi and Mekwunyei et al. in Nigeria identified social stigmatization about miscarriages, unmarried pregnant mothers and pregnant adolescents’ perceptions of stigmatisation to have a negative influence on early ANC visits, leading to inadequate ANC and SBD utilization services [82,167,172]. Dansou et al.’s study in the Benin Republic reported household wealth index, female education and desire for pregnancy as the most significant variables associated with meeting the recommended 4+ ANC and SBD utilization services [145]. Gravida and gestational age, long distance to the health facility, women without autonomy, and cultural beliefs/practices were reported by Konlan et al.’s study in Ghana, Uidbjerg et al. in Uganda and Fisseha et al. in Ethiopia as factors influencing low FANC utilization [44,116,122]. Mpembeni et al. and Olayinka et al.’s studies in Tanzania and Nigeria, respectively, identified poor awareness of maternal health services among women, dissatisfaction of mothers, poor resources, long distances to health facilities, a lack of means of transportation to health facilities, bad roads, and cost of health services as a major deterrence to ANC [88,157]. These reports are evidence that ANC and SBD utilization services need to scale up in SSA countries, where many pregnant women receive little or no ANC services during pregnancy, contributing to increasing maternal mortality. Which can be prevented. Moreover, adequate ANC utilization services, as recommended by the WHO, can help reduce these obstetric complications, leading to reduced maternal mortality.

#### 3.2.3. Rural–Urban Disparities

Several authors have established that ANC and SBD services are very beneficial for all pregnant women and postnatal mothers, and there are trends of urban–rural disparities in terms of healthcare access from their findings (Table 5). Boamah et al.’s study in Ghana identified biosocial factors such as wealth status and parity to contribute largely to the overall gap in ANC service utilization [175]. Eke et al. and Kebede et al.’s findings in Nigeria and Ethiopia, respectively, reported ignorance in rural communities, poor attitudes of health workers and the cost of services as barriers to antenatal and facility delivery service utilization [49,76]. According to a study by Fagbamige and Idemudia, the least educated women living in rural regions and impoverished expectant mothers made the least use of ANC services even though they were the ones who needed them the most [79]. Gebre et al. in Ethiopia reported inequities, low economic status, illiteracy, rural residence, no occupation, and less access to mass media as factors [45]. He et al.’s study in Zambia identified the importance of addressing socio-demographic inequalities such as women’s education, ethnic background, the wealth status of the household, parity, husband’s education, and exposure to mass media to help promote the utilization of ANC services [140]. Langa and Bhatta, Okoli et al., Ruktanonchai et al., Nwosu & Ataguba, and Selebano & Ataguba, Tareke et al. Idriss et al. and Rutarema et al.’s studies in Tanzania, Nigeria, Eastern Africa, Nigeria, Southern Africa, Ethiopia, Benin and Uganda reported on socio-economic inequalities among women, lower levels of education, poorer backgrounds and household wealth status as the most significant contributing factors to the gap between urban and rural areas in healthcare service utilization [52,56,83,85,109,117,143,156]. Overall, these findings show the disparities that exist in maternal health services in urban–rural communities, making progress towards reducing maternal mortality a huge challenge. In rural areas, there are fewer resources, fewer healthcare providers, and poor access to healthcare overall.

#### 3.2.4. The Impact of Intimate Partner Violence and Substance Abuse

The findings from the studies conducted in many countries highlighted a negative relationship between intimate partner violence (IPV), an early ANC visit and a minimum of four ANC utilization services. Women who experienced any form of IPV were less likely to meet the requirement of four basic ANC visits (Table 6). Bahati et al.’s study in Rwanda and Idriss et al.’s study in the Benin Republic stated the impact of IPV experience on ANC service utilization pointers including the following: the commencement of care within the first three months of gestation, receipt of at least four ANC visits, the updated WHO recommended eight-visit model (ANC-8), and receipt of care from skilled providers [136,143]. Bahati et al., Idriss et al., Ononokpono and Azfredrick and Ragetlie et al.’s studies in Rwanda, Benin, Nigeria and Togo, respectively, reported on married women living with their husbands who experienced physical, psychological and sexual violence [90,136,143,162]. Ononokpono & Azfredrick, in Nigeria, reported the prevalence rate to be 33.4% IPV, of which physical IPV was associated with low use of ANC [90]. Ndimbii et al.’s findings in Kenya also highlighted the effect of heroin drug use with unplanned pregnancy; fear of stigmatization from healthcare workers was a major factor that deterred this vulnerable group of women’s enrolment for ANC and SBD service utilization [103].

#### 3.2.5. Empowerment–Intervention Programme for Vulnerable Women

A study was conducted by Imo [80] to determine the impact of the independence of decision making by women on ANC institutional delivery services utilization; the study helped find out that decision-making autonomy for women significantly increased the chance of attending the recommended ANC visits. A survey was conducted in Nigeria, Guinea, Mali and Zambia by Kareem et al., and a link was found between women’s empowerment, fulfilling the WHO-recommended ANC model of eight or more visits and early ANC visits amongst pregnant women [98]. Obare et al.’s study in Kenya reported that poor women were less likely to use safe health facility delivery and skilled delivery care [104]. Onono et al.’s findings in Kenya reported on technology innovations such as a mobile phone and a 24 h transport navigation system to enhance maternal child health service utilization with interactive gestation-based text messages (MAccess) [105]. Bonfrer et al. and Sango et al.’s studies in Ghana and Gabon, respectively, reported that women with healthcare insurance coverage were more likely to use ANC and SBD services than those without a national health insurance scheme [126,149], while Seid and Ahmed’s study in Ethiopia revealed that more than 84% of these women lived in a rural area [51]. A study in Burundi was conducted by Bonfrer et al. to investigate the outcome of performance-based financing (PBF) on maternity and childcare utilization and quality [160]. They found that PBT improved the utilization of ANC visits, institutional delivery and the quality of most maternal and childcare but did not improve the targeting of unmet needs for ANC [160]. Ekirapa-Kiracho et al. discovered that in Uganda, interventions such as home visits by community health workers (CHWs), village health teams (VHTs), health education through radio spots, talk shows and quarterly community dialogues had a significant effect on the utilization of maternal and newborn services and care practices [120]. Lee et al.’s study in the Democratic Republic of Congo identified the distance and signboard (1.000) as the most important factor to be considered for 4+ ANC services in the communities [137]. In Malawi, Mamba et al. report that community members’ poverty (financial cost) was an issue [163]. The report states that most mothers do not have clothes to wrap their newborns in, and they do not have access to clinical services because of an inability to pay for the authorization of documents from village heads. This applies to women who do not have partners. This also undermined the use of ANC and SBD services. A study conducted in Ghana by Nuhu et al. reported the impact of T4MCH intervention on MCH service utilization and found that T4MCH improved ANC and skilled delivery service utilization in the intervention district [130]. Oguntunde et al.’s study found that male support groups and engagement are considered as being important to the health of women during pregnancy, labour, delivery and the postpartum period, as well as the health of newborns and children [84]. Their study found that male support had an overwhelming positive influence on maternal services utilization in the home and at the community level. Okonofua et al.’s study in Nigeria identified the cost of services and gender-related issues with poor ANC and other MHC services, especially for most women in rural communities [87]. Ruton et al.’s findings in Rwanda reported that the implementation of a rapid SMS program is important but not sufficient alone. Recommended rapid SMS combined with supervision, training, and the provision of equipment increases the use of maternal and child health services utilization [134].

#### 3.2.6. Climate Change and COVID-19 Crisis Barrier to ANC

Studies conducted by Galle et al. in the Democratic Republic of the Congo (DRC) and Banke-Thomas et al. in four SSA countries reported that the reasons for not seeking maternal healthcare during the COVID-19 pandemic crisis were to avoid COVID-19 vaccination, the high cost of transportation or lack of transportation, fear of not wanting to be infected in the hospital and service closures [39,178]. According to Scanlon et al., in Kenya, nationwide strikes by health workers had a negative influence on mother and child health service utilization [110]. Stone et al. in Mozambique discovered that connections existed between the rainy season and the levels of utilization of maternal healthcare [148]. Findings show that the rate of ANC visits and institutional deliveries during the rainy season was lower, leading to 74 maternal deaths, which could have been prevented if the mothers had access to health facilities.

## 4. Discussion

The importance of the ANC and SBD’s role in reducing maternal and infant deaths can be seen in the provision of delivering highly effective health interventions during critical periods. The prevalence of the low utilization of ANC and SBD service coverage in SSA countries, therefore, urgently requires policy attention to improve access, resources, rural–urban disparity, women’s literacy, awareness, utilization, and quality of maternal health services. Moreover, the consequences of maternal mortality do not only affect families alone but cause considerable distress to the community, the nation, and the world at large. Hence, ensuring the utilization of ANC and SBD services is critical to achieving positive pregnancy outcomes that can reduce maternal mortality and help to achieve the SDG 3.1 target by 2030. It is worth noting that most of the included studies were conducted in Ethiopia, followed by Nigeria, published between 2019 and 2023, and the majority of the studies adopted a cross-sectional design. Overall, the mean quality score was quite high, which indicates its robustness and reliability. Our findings highlighted various determinant factors such as access to the health facility, wealth status index, age group, marital status, women and their partners’ literacy, place of residence, media, support system, resources, health personnel and decision-making autonomy, associated with the utilization of ANC and SBD services and meeting the recommended ANC visits’ completeness. Additionally, geographical and socio-economic factors were also found to play a major role in utilising ANC services [32,33,123].

Based on the findings from this review study, low ANC and SBD are evident in this review. The study identified some contributing factors, such as poor/lack of access to healthcare, poverty, inequalities, educational level, and societal norms, which all play major roles in limiting the utilization of ANC services in SSA. It is worth noting that out of a total of 137 included studies, 92 reported low ANC and SBD utilization services. Furthermore, factors such as the poor awareness of ANC services, resource constraints, a shortage of manpower, poor/lack of infrastructures in the rural community, poor health system, bad roads, poor transportation system, and HCW attitudes were contributing to the low prevalence reported in this review [43,131,135,184]. To surmount these hurdles, a comprehensive intervention strategy must be adopted that encompasses a wide range of sectors. It demands the allocation of resources to advance healthcare infrastructure, equip healthcare professionals with in-service training, and the education of women and girls [164]. Education campaigns should be conducted to inform women of the benefits and importance of accessing ANC services, followed by SBD. Poly attention and effective intervention support services are required to improve the overall challenges to the utilization of ANC and SBD and contribute towards the SDG 3.1 target progress made so far. More needs to be performed in SSA to address the factors which are affecting this region.

The disparity between urban and rural areas is a reality. Many less privileged pregnant women in remote and rural areas could not access health facilities due to a lack of access to transportation, making it more difficult for them to access health services where they reside. This led to an overall poorer quality of life due to a lack of access to ANC and health screenings for pregnant women. Without intervention on access to ANC and SBD, rural pregnant women may continue to be susceptible to suffer from obstetric complications and deaths that could have been prevented through the provision of continuous good quality care. Furthermore, there is a disparity in health personnel proportions in urban–rural areas, which is hard to ignore, as there is often a shortage of nurses, midwives, and doctors in rural areas. Strategies to attract and retain health workers in remote and rural areas could help reach the least privileged women. For example, the mean for health personnel among the poor in most SSA countries is 32% compared to the rich at 84% [185]. This study suggests that the above factors should be carefully considered when developing strategies to increase ANC and SBD utilization services in SSA. Also, empowering women through economic strengthening opportunity schemes education, and appropriate cultural services can promote social inclusion and reduce inequality, which can lead to better health outcomes pre-, intra- and post-pregnancy. The challenge of accessing health facilities makes it difficult for women to receive proper ANC services or give birth under the supervision of doctors, midwives, and nurses in some communities. Regarding where and when medical facilities are available, geographical locations are a hindrance to accessibility to these women due to insufficient healthcare infrastructure. The disparities between rural and urban populations are exacerbated a by lack of investment in the public health sector by the government through poor resource allocation, and even when allocated, these resources are looted or even underutilised. These disparities not only cause struggles with financial constraints but also failure to retain qualified personnel. The ratio of healthcare workers (HCWs) falls below the standard of the international ratio, which could lead to work overload on HCWs, hence the poor quality of service delivery in the majority of health facilities [186]. For example, access to quality ANC services can help to detect and reduce the risk of complications during pregnancy, while social support can help to reduce maternal stress and provide psychological support. There is a need to build more health facilities, improve the existing healthcare systems, recruit more HCWs and strengthen support services. Governments, policymakers, and other key stakeholders should consider investing more in the healthcare system, ensuring that essential services are available, accessible and affordable to all reproductive-age women, particularly in rural areas.

Intimate partner violence and substance abuse’s impacts on the utilization of ANC visits were a recurring theme in this review. Evidence points to the physical and psychological experience of these pregnant women. Women who experienced intimate partner violence or substance abuse had lower rates of ANC visits and were more likely to experience delayed or late ANC visits. Additionally, these women were more likely to have high-risk pregnancy complications. It is recommended that the government should prioritise integrating strategies, policy and evidence-based interventions to address this challenge.

The widespread occurrence of the COVID-19 pandemic crisis has made the situation even more complex. There is a need for interventions to help improve the situation and achieve the SDG 3.1 target by 2023. More research is needed on how to strengthen professional and healthcare system competence to improve quality services, climate change and COVID pandemic preparedness, and responses in SSA countries, as their unique challenges should not be overlooked.

An important strength of this review is that it covers a wide range of the literature on the utilization of ANC and SBD services in SSA. Mapping this research evidence serves as a valuable resource for researchers, policymakers, and health professionals. Additionally, the review highlighted studies with high overall quality scores of (88% ± 14), which enhances the credibility and reliability of our findings. Having two reviewers assess the same data makes the results of this study more reliable and accurate since it allows the reviewers to independently evaluate every study and identify any discrepancies or errors that may have been overlooked by one. A key contribution of this study is its ability to identify key themes based on the trends in the literature and important gaps in the existing research, which can be used to inform government policies and future studies. Despite the strength of this study, it has potential limitations. The fact that all the studies are published in English may not reflect the diversity of the research population. Furthermore, studies on mother and child healthcare service utilization are restricted, with more than half coming from the demographic health survey and all being cross-sectional. Further study on maternal health service utilization in SSA regions where the burden of maternal mortality is highest is required to fill the research gaps.

## 5. Conclusions

This scoping review highlights the potential evidence of and impacts on ANC and SBD service utilization for meeting the SDG 3.1 target. The review emphasises the importance of addressing urbanization, resource constraints, wealth status index, women’s literacy, awareness, and socio-economic and cultural norms, which were associated with poor ANC and SBD service utilization. Intervention strategies and policy development are needed to improve female education, health facility access/utilization, socio-economic and the quality of reproductive health services. Further research is recommended to target other bottlenecks of maternal health service utilization.

## Figures and Tables

**Table 1:** Eligibility criteria for study selection using PCC framework.

Eligibility Criteria	Inclusion Criteria	Exclusion Criteria
Population	Pregnant women and postnatal mothers.	
Concept	Antenatal care (ANC) and skilled-birth delivery (SBD) service utilization. The use of ANC services refers to the availability and use of healthcare during the gestation period, allowing pregnant women to obtain substantial interventions for their own and their child’s health. Skilled birth delivery services are when pregnant women give birth in a health facility with the help of a midwife, nurse or medical personnel.	
Context	Studies conducted in SSA (countries in the WHO Africa Region)	Studies focused on other regions of WHO, such as Southeast Asia, the Western Pacific, Europe, and the Eastern Mediterranean region
Study design	Original research that employed quantitative and qualitative studies and mixed methods.	Review papers such as a literature review, narrative review, rapid review, and expert review; papers with only abstracts; and editorial comments.
Publication types	Peer-reviewed publications	Grey literature such as theses, dissertations, and conference papers.
Timeframe	Publications within 10 years from 2012 to 2023.	
Language	All publication languages were considered for this study.	

**Table 2:** Characteristics and quality appraisal of the included studies.

Characteristics	Number (*n*) Percentage (%)	References to the Source
**Study country**		
Ethiopia	40 (29.2)	[31,32,33,34,35,36,37,38,39,40,41,42,43,44,45,46,47,48,49,50,51,52,53,54,55,56,57,58,59,60,61,62,63,64,65,66,67,68,69,70]
Nigeria	30 (21.9)	[38,71,72,73,74,75,76,77,78,79,80,81,82,83,84,85,86,87,88,89,90,91,92,93,94,95,96,97,98,99]
Kenya	18 (13.1)	[38,99,100,101,102,103,104,105,106,107,108,109,110,111,112,113]
Uganda	16 (11.7)	[39,41,91,94,95,99,109,112,114,115,116,117,118,119,120,121]
Ghana	18 (13.1)	[38,41,91,95,96,99,113,121,122,123,124,125,126,127,128,129,130,131]
Rwanda	10 (7.3)	[91,94,99,109,113,132,133,134,135,136]
Democratic Republic of Congo	10 (7.3)	[38,41,52,91,94,99,118,121,137,138]
Zimbabwe	11 (8.0)	[38,41,52,91,94,96,97,99,113,131,139]
Zambia	12 (8.8)	[38,41,52,91,94,96,97,98,99,113,140,141,142]
Benin	05 (3.6)	[99,121,143,144,145]
Guinea	05 (3.6)	[91,98,99,146,147]
Mozambique	06 (4.4)	[41,52,91,94,99,148]
Gabon	03 (2.2)	[41,91,149]
Mali	09 (6.6)	[38,41,91,94,96,97,98,118,150]
Sudan	01 (0.7)	[151]
Burkina Faso	06 (4.4)	[38,94,99,113,152,153]
Namibia	06 (4.4)	[41,52,96,99,113,154]
Tanzania	10 (7.3)	[41,52,99,109,113,155,156,157,158,159]
Botswana	01 (0.7)	[52]
Burundi	07 (5.1)	[91,96,97,99,109,113,160]
Togo	06 (4.4)	[38,91,97,113,161,162]
Cameroon	05 (3.6)	[38,91,96,99,113]
Malawi	11 (8,0)	[52,81,91,96,99,163,164,165,166,167]
Sierra Leone	05 (3.6)	[38,41,91,113,168]
Somalia	03 (2.2)	[94,169,170]
Liberia	07 (5.1)	[38,41,94,97,99,113,171]
Senegal	04 (2.9)	[94,99,113,172]
Cote d’Ivoire	05 (3.6)	[41,94,97,99,113]
Niger	03 (2.2)	[52,99,113]
Angola	03 (2.2)	[52,97,99]
South Africa	04 (2.9)	[52,97,99,113]
Lesotho	04 (2.9)	[52,97,99,113]
**Year of publication**		
2013–2018	53 (38.6)	[33,35,40,44,45,57,59,63,65,66,67,68,69,71,74,79,81,86,88,90,92,96,101,103,104,106,107,108,109,114,117,120,127,128,131,132,134,135,138,139,141,145,151,160,161,163,166,173,174,175,176]
2019–2023	84 (61.3)	[31,32,36,38,39,41,42,43,46,47,48,49,50,51,52,53,54,56,60,61,62,64,72,73,75,76,77,78,80,82,83,84,85,87,91,93,94,95,96,97,98,99,100,102,105,110,111,112,113,116,118,119,121,122,123,124,125,129,130,133,136,137,140,141,143,144,146,147,148,149,150,152,154,156,157,162,164,169,170,171,172,177,178,179,180,181]
**Study design**		
Cross-sectional study	119 (86.8)	[31,32,33,34,36,37,38,41,42,43,44,45,46,47,48,71,72,73,74,75,76,78,79,80,83,85,86,87,88,90,91,93,94,95,96,97,101,102,104,106,118,119,120,123,124,125,126,127,128,129,130,131,132,136,137,140,143,145,147,150,160,161,163,166,175,178,179,181,182,183]
Qualitative study	11 (8.0)	[77,84,100,103,105,116,124,139,141,151,154]
Cohort study	04 (2.9)	[49,58,74,110]
Mixed methods study	03 (2.2)	[39,53,92]
**Study population**		
Pregnant women and postnatal mothers	124 (90.5)	[41,42,43,44,45,46,47,48,49,50,58,74,76,77,78,79,80,81,82,83,84,85,86,87,88,100,101,102,103,104,105,106,110,114,116,118,120,122,126,127,128,129,130,131,132,133,137,139,140,141,145,151,154,156,160,163,172,178,179]
Migrant mothers	01 (0,7)	[161]
Adolescent and youth	06 (4.4)	[38,82,118,119,154,166]
Women with drug abuse	01 (0.7)	[103]
Women living in slums	01 (0.7)	[107]
Women with IPV	04 (2.9)	[90,136,143,162]
**Quality appraisal score**		
Between 50 and 75%	50 (36.5)	[48,50,81,83,85,86,87,88,102,104,106,114,118,129,130,133,140,163,178]
75% and above	87 (63.5)	[45,46,47,49,58,74,77,80,82,84,90,100,103,105,107,110,116,122,128,132,137,139,141,143,151,154,156,166,172]

**Table 3:** Determinant of ANC and SBD service utilization in SSA.

Location of the Study	Author and Year	Findings
Nigeria	Adewemimo et al., 2013 [71]	Staffing of medical personnel: nurses, midwives and doctors in a health facility. Health system and costs should be met through maternal health fee services. Girl’s education should be promoted. Encouraging members of the male gender to actively participate in maternal child health.
Ethiopia	Abebe et al., 2019 [91]	Follow-up to encourage women through education strategies during ANC significantly increased access and improved the number of antenatal care visits and utilization
Ghana	Afaya et al., 2020 [123]	A major factor that influenced ANC visits was the National health insurance scheme.
Ethiopia	Afework et al., 2014 [33]	Antenatal care attendance at least four times during pregnancy was significantly associated with visits by Health Extension Workers [odds ratio 3.46(95% CI 3.07, 3.91)
SSA	Ahinkorah et al., 2021 [95]	Pregnant women who were given permission seemed to have timely antenatal care visits. Women who benefitted from treatment funding seemed more inclined to attend the recommended number of antenatal care appointments (aOR = 1.38, 95% CI = 1.11–1.73) Lack of funds for treatment, distance to the health facility or desire to travel alone were major determinants hindering antenatal care visits and lack of access to permission; this was the situation in Guinea, Zambia and Mali.
Guinea	Ahinkorah et al., 2021 [147]	Women aged 15–24, women in education, partners with education, the richest wealth quintile women, planned pregnancies, Muslim women, and those who took healthcare decisions alone and listened to the radio had higher odds of antenatal care uptake. Level of education was a major factor that influenced ANC and SBA service utilization.
Ethiopia	Alemayehu et al., 2020 [34]	Some of the significant predictors of ANC 4+ utilization include living in an urban environment, access to electronic media devices and having 2–5 children.
Ghana	Alhassan et al., 2020 [124]	Interventions in the health system at the community level are needed. High-facility-based SVD and child immunization data corresponded with high ANC visit records. Efficient tactics for inspiring and retaining a frontline medical health workforce led to increased ANC enrolment. Financing of universal coverage for quality ANC services improved the potential of service utilization.
Ghana	Ameyaw et al., 2021 [125]	Only 21.2% utilised all three components of MCH. Women with National Health Insurance Scheme (NHIS) cover utilised ANC and other components of MCH more. Married women and wealth status.
Angola	Amu et al., 2023 [97]	The prevalence of ANC was 58%. Health insurance coverage is significantly associated with increased utilization of ANC services.
Togo	Atake, 2018 [161]	ANC visits were higher amongst migrant household mothers than non-migrant mothers. The welfare of migrant households could not be com-pared to that of non-migrant households, as the migrant households had better welfare. Access to health insurance gives more financial protection to migrant mothers.
Uganda	Atuhaire et al., 2020 [180]	Increased early ANC utilization is directly associated with ages 35–49, education, no distance issues to a health facility, the costs of health services, availability of community workers and desire for pregnancy.
Uganda	Atuoye et al., 2020 [119]	Being a primigravida was more likely to meet all three maternal health service indicators. Important factors linked with ANC visits and SBAs are literacy, affluence, autonomy. power and the distance of residence to the health centre.
Nigeria	Adedokun et al., 2023 [93]	Significant predictors for SBA utilization services are the educational level of women, husband’s occupation, pregnancy complications and place of previous childbirth. Poverty rate, lack of medical equipment supplies, and absence of healthcare providers are identified barriers to SBA utilization. Enabling factors are access to medical staff, husband’s support and the cost of services.
Congo Egypt and Ghana Nigeria and Zimbabwe	Dimbuen et al., 2018 [131]	There is a clear socio-economic stratum among reproductive-age women using maternal health service utilization. Households’ status, education and access to health facilities were positive factors associated with antenatal care and skilled birth attendant delivery.
SSA	Adedokun et al., 2020 [94]	Women aged 25–34 years with education and a wealth status index adequately utilised ANC services.
SSA	Bain et al., 2022 [38]	Wealth status increased the probability of utilising ANC and other maternal healthcare services.
SSA	Budu et al., 2021 [41]	ANC visits are significantly associated with complete children.
Ethiopia	Birmeta et al., 2013 [40]	The percentage of women who attended at least one antenatal visit during their last pregnancy was 87%. The attendance of ANC services was largely influenced by certain demographic variables like age, level of education, income, exposure to the media and knowledge of the danger signs of pregnancy.
Ghana	Esena & Sappor, 2013 [127]	A large percentage of respondents, 37 (94.1%), attended ANC. Maternal education, occupation (Job type), wealth status and religion were statically related to the utilization of skilled delivery.
Ethiopia	Gebrekirstos et al., 2021 [46]	The following was reported as the determinants of adequate ANC service utilization: education, peer influence, husband support, wealth status index, follow-up strategy, history of risky pregnancy, and planned pregnancy.
Ghana	Gudu & Addo et al., 2017 [128]	Of 400 women, 97.3% received antenatal care at their last pregnancy, while 75.0% of them had four or more ANC visits. Pregnancy planned (aOR = 3.9; 95% CI: 1.8–8.3) and awareness of danger signs in pregnancy.
SSA	Iacoella & Tirivayi, 2019 [118]	Paired education for female and male partners was categorically associated with antenatal care. The utilization of all types of maternal healthcare services can be linked to wealth and access to maternal information from the media.
Ethiopia	Kotiso et al., 2020 [50]	In total, 34.5% received at least one antenatal care visit for the current pregnancy. Factors associated with antenatal care utilization included food security, education attainment, good level of knowledge of antenatal care and being from a wealthy household.
Ethiopia	Geda et al., 2021 [47]	Female education, parity, experience of terminated pregnancy, residing in more affluent households and polygamous families indicated positive impacts on ANC visits and a strong effect on institutional delivery service utilization.
Nigeria &Malawi	Kuuire et al., 2017 [81]	Wealth status strongly influenced ANC visits as well as the timing of the first visit in Nigeria but not in Malawi.
Kenya	Owili et al., 2016 [106]	Significant association between adequate utilization of ANC and family structure in terms of monogamous (OR= 1.84) and polygamous (OR = 1.72) families and formerly married (OR= 1.84) compared to unmarried women.
Nigeria	Okonofua et al., 2018 [86]	Religion and faith
Kenya	Owiti et al., 2018 [107]	Components that helped free maternal service uptake were a positive view of the public health facility, proximity to the health facility, learning about the program from a support group and a swift wait time for doctor’s examinations.
SSA Ethiopia, Nigeria, Ghana, Benin, Kenya, Rwanda, Burkina Faso Burundi, Cameroon Chad, Comoros, Congo, Tanzania, Uganda, Zambia, Democratic Republic of Congo, Republic of, Cote d’Ivoire, Gambia, Lesotho, Liberia, Madagascar Malawi, Mali, Mozambique, Namibia, Niger, Senegal, Sierra Leone, South Africa, Togo, Zimbabwe	Rosser et al., 2022. [121]	Increase in manpower resources had a positive influence on maternal health service utilization, which included ANC and facility birth attendance. HCW densities are associated with an increased likelihood of ANC utilization services.
Malawi	Rai et al., 2013 [166]	Maternal age, household economic status, and status of the child were reported to positively influence at least four antenatal care visits.
Ethiopia	Semagn, 2023 [53]	The significant statistics which are characteristically linked to health facility delivery are educational status, wealth index, marital status, attending ANC in the first trimester of the gestation period and access to an ANC-trained provider.
Guinea	Shibre et al., 2021 [146]	In Guinea, factors that influence the utilization of skilled ANC services are exposure to media, decision-making power, maternal husband education status, economic status and place of residence.
Malawi	Stewart & Hall, 2022 [164]	Inadequate ANC utilization service: only 24% of women received the recommended ANC package. The wealth index (OR = 1.33, 95% CI = 1.08–1.65), planned pregnancy (OR = 1.3, 95% CI = 1.11–1.51), and decision-making control (OR = 1.09, 95% CI = 0.80–1.49) all increased service utilization.
Ethiopia	Tarekegn et al., 2014 [57]	Thirty-four percent of women had ANC visits. Utilization of ANC services is more common among more autonomous women. Factors that influence the utilization of maternal health services are as follows: women’s education, place of residence, ethnicity, parity, women’s autonomy and household wealth.
Ethiopia	Teklesilasie & Deressa, 2018 [58]	At least one antenatal care visit was reported by women when husbands accompanied them 6. 27 times (95% confidence interval: 4.2, 9.3).
Ethiopia	Tesfaw et al., 2018 [59]	Mother’s age, urban residing areas, and distance no more than 16–30 min from a health facility were factors influencing the use of skilled delivery practice. Mothers with four or more antenatal care (ANC) visits and knowledge about pregnancy complications also utilised skilled delivery services.
Ethiopia	Tesfaye et al., 2019 [60]	Important risk factors for SDC are as follows: educational history, knowledge of maternal health, prior use of skilled delivery care, place of residence and peer influence. Skilled delivery care utilization was strongly related to attendance of antenatal care services and pregnancy intention.
SSA	Tesssema et al., 2021 [113]	The frequency of recommended antenatal care utilization in sub-Saharan African countries was 58.53% [95% CI: 58.35, 58.71], with the Southern Region of Africa having the highest ANC utilization (78.86%) and the Eastern Regions having the lowest (53.39%). Place of residence, mother/husband educational level, maternal occupation, healthcare decision autonomy, wealth index, media exposure, access to healthcare, desired pregnancy, and birth order were all factors influencing recommended ANC utilization in Sub-Saharan Africa.
Ethiopia	Tiruneh et al., 2022 [62]	Family conversation during pregnancy and the delivery by caesarean birth notified to Health Extension Workers were predictors of the continuum of care.
Ethiopia	Tsegay et al., 2013 [63]	The percentage of 54% was the demography of the population of women who received ANC for their recent baby delivery. Marital status, education, husband’s occupation, and proximity of health facility to the village are factors that contributed to ANC utilization.
Ethiopia	Tsegay et al., 2021 [64]	Prevalence of antenatal care and institutional delivery care utilizations were 69.1% and 52.1%, respectively. Planned pregnancy, educational level, household training, middle wealth and richest wealth quantile were reported as positive factors associated with antenatal care utilization. The level of education of the spouse and ANC attendance was connected with institutional delivery
Ethiopia, Ghana, Nigeria, Zambia, South Africa, Tanzania, Kenya, Uganda, Rwanda, Burundi, Democratic Republic of Congo and South Sudan:	Wong et al., 2022 [112]	ANC utilization was high (>85%), and facility-based childbirth ranged widely at 77–99%. Cotonou and Accra outdid Nairobi and Ndjamena which had the lowest result. Most cities had inconsistent levels of utilization across the maternal CoC.
Ethiopia	Yadeta, 2018 [68]	Turnout for at least one antenatal care visit [AOR = 2.83; 95% CI (1.62, 4.93)] and baby delivery at a health facility [AOR = 3.31; 95% CI (1.67, 6.53)] were connected with significant knowledge of neonatal danger signs.
Ethiopia	Yeneneh et al., 2018 [67]	Those who were most likely to use ANC services were mothers with the highest wealth quintiles, lowest birth order, urban residence, younger age and higher education level.
Ethiopia	Zelalem et al., 2014 [66]	The demography of women who attended one ANC service during their previous pregnancy is 86.1%. In total, 61.7% received less than the recommended four visits while 46.2% commenced ANC in the second trimester. Out of 86.1%, only 25.3% gave birth in health institutions. Institutional delivery was used by local women at a lower rate (20.9% vs. 35.9 for urban women).

**Table 4:** The prevalence of low ANC attendance and associated factors.

Location of the Study	Author and Year	Findings
Nigeria	Abimbola et al., 2016 [92]	Constraints to the utilization of ANC services include funding, distance from health facilities, long wait times, negative attitudes of health staff and lack of authorization from spouses. The level of education and job status are significantly related to inadequate utilization of ANC.
Nigeria	Adewoyin et al., 2022 [72]	The prevalence rates of the recommended minimum ANC visits in Nigeria were low at 42.1% and 30.0% and lowest in the northern regions. Gender-related policies could improve maternal healthcare outcomes and services.
Ethiopia	Afework et al., 2014 [33]	HEW visits during pregnancy improved the utilization of maternal health services.
Nigeria	Akinyemi et al., 2018 [74]	Evidence of infant mortality was linked to poor use of ANC.
Burkina Faso, Cote d’Ivoire, Ghana, Liberia, Mali, Niger and Nigeria and Ethiopia, Kenya, Tanzania, Uganda, and Zambia	Amouzou et al., 2022 [181]	The COVID-19 pandemic had a negative impact on service utilisation of ANC-1 and ANC-4 in most SSA countries.
Ethiopia	Arefaynie et al., 2022 [36]	In total, 43.11% of women utilised antenatal care during their current pregnancy. Living in rural areas, poverty, being uneducated and single motherhood are associated with a low number of ANC visits.
Kenya	Riang’a et al., 2018 [108]	In total, 10% of the women booked before 13 weeks and illness in index pregnancy was their main reason for early booking and only 45% made four or more visits. The use of both biomedical and traditional antenatal care services was prevalent.
SSA Ethiopia, Nigeria, Ghana, Benin, Kenya, Rwanda, Burkina Faso Burundi, Cameroon Chad, Comoros, Congo, Tanzania, Uganda, Zambia, Democratic Republic of Congo, Republic of, Cote d’Ivoire, Gambia, Lesotho, Liberia, Madagascar Malawi, Mali, Mozambique, Namibia, Niger, Senegal, Sierra Leone, South Africa, Togo, and Zimbabwe	Rosser et al., 2022. [121]	Increase in manpower resources had a positive influence on maternal health service utilization, which includes ANC and facility birth attendance. HCW densities are associated with an increased likelihood of ANC utilization services.
Ethiopia	Ayele et al., 2019 [37]	Only 29.2% of women had skilled birth attendance (SBA) during their childbirth. The issues linked with SBD utilization include the education level of mothers, joint decision of couples on delivery location, ANC visit frequency, place of abode, birth preparedness and complication readiness status, as well as an understanding of obstetric danger indications after delivery
Kenya	Ayodo et al., 2021 [100]	Pregnancies not planned for also resulted in poor uptake of antenatal care (ANC) services. Limited knowledge, poor support system and poor government infrastructure. Work attitudes by healthcare practitioners, poor management of high-risk pregnancies and meagre resources at the health facilities were challenges to ANC service utilization by women.
Nigeria	Ajayi and Akpan, 2020 [73]	A low level of education was associated with the unlikeliness of pregnant women delivering a baby at a health facility. The likelihood of urban women giving birth at a health centre is twice that of rural women.
Mali	Bain et al., 2022 [150]	Prevalence of maternal healthcare utilization was 45.6% for ANC4+. At the individual level, ANC4 + utilization increased with increasing maternal age, formal education, and wealth status. Listening to the radio and watching TV were associated with increased maternal healthcare utilization. Cohabitation, women who considered obtaining money for treatment and distance to the health facility were big problems to ANC service utilization.
Kenya	Chorongo et al., 2016 [101]	Among the women who were aware of FANC, only 27% utilised its services. Departmental disharmony, long waiting hours, and unavailability of services when visiting the facilities led to dissatisfaction among mothers.
Benin Republic	Dansou et al., 2017 [145]	Household wealth index, female education and desire for pregnancy were the most significant variables associated with meeting the recommended four ANC services.
Ethiopia	Defar et al., 2021 [43]	In total, 39% (95% CI: 35 to 42) of women went for four or more prenatal care visits, while 55% (95% CI: 51 to 58) gave birth in health facilities. Frequent prenatal care visits and hospital deliveries can be associated with a higher wealth index.
Ethiopia	Dadi et al., 2019 [42]	Inadequate staffing of skilled health workers and limited healthcare supplies were a major hindrance to maternal health service utilization.
Ethiopia	Fisseha et al., 2017 [44]	Some of the predictors of skilled delivery services utilization are as follows: proximity to health facilities, women’s perceptions of the availability of adequate equipment, experience with birthing complexities, antenatal care, lower birth order and having an educated partner.
Nigeria	Etokidem et al., 2022 [78]	Lack of formal education is statistically associated with poor antenatal clinic attendance (AOR = 0.510, 95% CI = 0.219–1.188).
Rwanda	Hitimana et al., 2018 [132]	Cohabitating and single/unmarried were significantly associated with lower HRQoL. Educational level on HRQoL was statistically significant as well.
Uganda	Kawungezi et al., 2015 [114]	ANC awareness from health workers’ role (72.04%), the media (15.46%) and friends (12.50%). Health facility distance, husband’s decision, and “the availability and involvement of TBA, a wrong opinion during pregnancy about ANC, poor financial support and being economically constrained” were reported.
Senegal	Kim et al., 2019 [172]	Social stigmatization about miscarriage negatively influences early ANC utilization services. Social stigma towards unmarried mothers causes them to hide their pregnancy, leading to inadequate ANC utilization. Husband’s decision and social support affect ANC utilization.
Ghana	Konlan et al., 2020 [122]	Proximity to health institution and permission to use FANC were substantially linked with poor utilization (*p* < 0.001). Fear of witchcraft was associated with decreased FANC use (*p* < 0.001)
Rwanda	Kpienbaareh et al., 2022 [133]	Pregnant women who were not aware of pregnancy problems were less likely to seek ANC services during the first trimester (odds ratio [OR] = (0.76, *p* < 0.01) and meet the WHO’s recommended minimum of eight visits (OR = 0.66, *p* < 0.01)
Tanzania	Mpembeni et al., 2019 [157]	Only 34.4% are aware of their right to access maternal health services. Occupation and education level showed a statistically significant association with awareness of access rights.
Nigeria	Mekwunyei & Odetola, 2020 [82]	Teenagers who are pregnant reported a mean ± SD of 3.4714 for their perception of stigma. Education, unmarried teenagers, availability/accessibility of MHS facilities, the cost of MHS [*p* = 0.001] and coercion/violence from partners [*p* = 0.000] were statistically significant for the utilization of MHS and maternal services.
Somaliland	Mouhoumed & Mehmet, 2021 [169]	Fewer antenatal care visits are significantly associated with age, gravida, and gestation age. Early marriage and large family size are associated with delay in the commencement of the first antenatal care visit, and the recommended four visits
Kenya	Mutai & Otieno, 2021 [102]	In total, 37.3% of pregnant women do not utilise FANC services. Educational level, occupation and income, the facility, and the waiting time significantly influence the utilization of FANC services
Ghana	Numah et al., 2019 [129]	Low level of knowledge of mothers about pregnancy emergencies. Socio-economic characteristics and healthcare access influenced the utilization of maternal healthcare.
Namibia	Shatilwe et al., 2022 [154]	Distance to the nearest clinics was amongst the leading challenges affecting accessibility and utilization of MCHI for pregnant adolescent girls. Also, poor support, transport fares, poor road infrastructure and non-availability of transport were key barriers to accessibility and utilization of clinic services.
Nigeria	Olayinka et al., 2014 [88]	Lack of understanding, a negative obstetrics history, provider attitudes, availability, accessibility and husbands’ decisions are some of the many challenges women face when trying to use maternal health services.
Nigeria	Okonofua et al., 2018 [86]	Reasons for non-use of PHCs for antenatal were as follows: long distances to PHCs, high costs of services and poor quality of PHC service delivery.
Kenya	Rosser et al., 2014 [176]	Non-Kikuyu women in Nairobi who are less educated, poorer and those living farthest from public health services had lower ANC visits and delivered more frequently outside of health institutions. During pregnancy, approximately 54% of pregnant women did not attend the recommended four ANC visits during gestation.
Rwanda	Rurangirwa et al., 2017 [135]	About 54% of pregnant women did not make the recommended four visits to ANC during pregnancy. Poor utilization of ANC services was higher for older age women, 31 years and above, single women and women with poor social support. Women aged 31 years or older, single women and women with poor social support had poor utilization of ANC services.
Uganda	Rutaremwa et al., 2015 [117]	Women with education from the richest households utilised maternal healthcare packages. The use of modest maternal healthcare services is less likely amongst women who live in rural areas, Muslims and those who are married.
Cameroon, Democratic Republic of Congo, Liberia, Malawi, Mali, Nigeria, Sierra Leone, and Somalia	Shapira et al., 2021 [54]	Outpatient consultation services were mostly affected by decreases in antenatal care service utilization, which was detected in some countries
Uganda	Uldbjerg et al., 2020 [116]	Absence of support from a spouse, inadequate care quality, negative attitudes among health personnel, and cultural behaviours not being aligned with ANC are just some of the barriers to ANC utilization. Procedures at health institutions and institutional structures, including mandatory HIV tests, material requirements, and transportation barriers, made it impossible for some pregnant women to attend ANC services.
Ethiopia	Tareke et al., 2021 [56]	High likelihoods of underutilization of FANC exist among mothers who are not exposed to media, reside far from the health facility, and lack companionship and ethnicity.
Ethiopia	Umer et al., 2020 [170]	Only 27% [95% CI 22.8–31.2%] of women used ANC services, whereas 22.6% [95% CI 18.7–26.5%] obtained skilled delivery services. In total, 43% of respondents were ignorant of ANC, and 46% did not consider delivery at a health facility necessary. The determinants of antenatal care utilization are the husband’s educational status, the attitude of the women towards healthcare services and funding support from husbands: all these have substantial impacts on antenatal care utilization. Healthcare worker’s attitudes, women’s perceptions of institutional delivery, antenatal care utilization and no maternal healthcare fees were associated with skilled delivery service utilization.
Kenya	Wairoto et al., 2020 [111]	The coverage of ANC4 across sub-counties was low, with 17% in the Mandera Western sub-county. Low socio-economic status, maternal education, marital status, age at first marriage, and birth order were all associated with ANC utilization.
Ethiopia	Yaya et al., 2019 [171]	Maternal education, exposure to media and wealth index all influence women’s use and attendance rate of ANC visits. However, women in rural areas had lower attendance rates for ANC visits and formal institutional delivery.

**Table 5:** Rural–urban disparities.

Location of the Study	Author and Year	Findings
Ghana	Boamah et al., 2016 [175]	Biosocial factors such as wealth status and parity contribute largely to the overall gap in ANC service utilization.
Nigeria	Eke et al., 2021 [76]	The quality of care is attributed to good utilization of maternal health services both in urban and rural communities. Also, in rural communities, ignorance, poor attitudes of health workers, and cost of services are a barrier to antenatal and facility delivery services utilization.
Nigeria	Fagbamigbe & Idemudia, 2017 [79]	The use of ANC was generally lower among the poor and the least educated women living in rural areas who needed ANC the most.
Ethiopia	Gebre et al., 2018 [45]	Wealth-related inequalities were significantly high in 2016. Limited access to mainstream media, unemployment, rural residency, illiteracy, and low socio-economic position were issues that caused inequities.
Zambia	He et al., 2021 [140]	Addressing important socio-demographic inequalities such as women’s education, ethnic background, wealth status of the household, parity, husband’s education, and exposure to mass media in using maternal healthcare services may help promote the utilization of ANC service.
Tanzania	Langa and Bhatta, 2020 [156]	Socio-economic inequalities among women in maternal healthcare, with lower levels of education and household wealth, are significantly wider in rural than urban areas.
Ethiopia	Kebede et al., 2021 [49]	Pregnant women who reside in rural regions had a higher risk of developing MNM than those who reside in cities with an adjusted hazard ratio (AHR) of 1.68 (95% CI, 1.01, 2.78).
Nigeria	Nwosu & Ataguba, 2019 [83]	At least four ANC visits (CI = 0.582) and a higher number of ANC visits (CI = 0.357) were disproportionately concentrated among the rich. In the rural communities, widespread disparities with a high poverty level were prevalent.
Nigeria	Okoli et al., 2020 [85]	There is a significant gap in the utilization of FBD between urban and rural areas and well-educated and wealthier mothers. Six geopolitical zones are reducing this gap by 7.8% and 1.8%, respectively. This is according to the Theil index, which states that there are relative inequalities in ANC and FBD.
Burundi, Kenya, Rwanda, Tanzania, and Uganda.	Ruktanonchai et al., 2016 [109]	A reduced likelihood of receiving MNH care by women across all outcomes with income index and education level as major determinants.
SSA	Samuel et al., 2021 [99]	The disparity in healthcare service utilization between urban and rural locations is often caused by socio-economic factors like household wealth index, exposure to media and the educational level of women and their husbands.
Botswana	Selebano & Ataguba, 2022 [52]	Women from poor families attend fewer ANC visits than those from a wealthy background. Wealth status, education, and the number of children were the socio-economic inequalities in ANC coverage in SADC.
Uganda.	Rutaremwa et al., 2015 [117]	There is a high desire for maternal healthcare package utilization services among women with education and wealth status index compared to those who have no education. Women living in the rural community and from the Muslim faith were less likely to moderate maternal healthcare services.
Ethiopia.	Tarekegn et al., 2014 [57]	In total, 34% of women had ANC visits, and 11.7% used skilled delivery attendants. The utilization of ANC and skilled delivery attendant services is more common among women with education, women who reside in urban areas, and women with autonomy and wealth status index.

**Table 6:** The impact of intimate partner violence and substance abuse.

Location of the Study	Author and Year	Findings
Rwanda	Bahati et al., 2021 [136]	In total, 17% of married women living with their husbands reported physical violence, 22.8% reported psychological violence and 9.2% reported sexual violence. Physical IPV has a substantial adverse connection with both early and adequate ANC.
Kenya	Ndimbii et al., 2018 [103]	Women’s access to essential services across the RMNCH continuum was low. Unpregnant due to amenorrhea effect of drug use, stigma from healthcare workers, and long waiting times were major factors preventing women’s utilization of existing RMNCH services. The misplacement of priorities to spend money on heroin rather than health-related costs also deterred enrolment for antenatal care services.
Benin Republic	Idriss et al., 2021 [143]	Women who never experienced IPV (OR 0.753, 95% CI: 0.628–0.901; *p* = 0.002) had 25% less odds of accessing the basic four ANC visits. Also, the wealth index quintile, making decisions on household and healthcare, and having a paid job increased the chances of utilising four ANC services. Islamists were less likely than other faiths to use ANC.
Nigeria	Ononokpono & Azfredrick, 2014 [90]	Prevalence rate of 33.4% IPV. Physical IPV was associated with the low use of ANC. Emotionally abused women were less likely to seek competent child delivery support.
Togo	Ragetlie et al., 2020 [162]	Women who have been through some aspects of IPV were less likely to meet all of ANC utilization criteria. Socio-economic variables such as education and household wealth all contribute to disparity in healthcare service utilization.

## Data Availability

Data for this study were sourced from secondary data and are available on request.

## Supplementary Materials

The following supporting information can be downloaded at: https://www.mdpi.com/article/10.3390/ijerph21040440/s1, PRISMA-ScR checklist.
